# An Open Prospective Study on Whether Intracytoplasmic Morphologically Selected Sperm Injection (IMSI) Offers a Better Outcome Than Conventional Intracytoplasmic Sperm Injection (ICSI)

**DOI:** 10.7759/cureus.19181

**Published:** 2021-11-01

**Authors:** Amr Moubasher, Tarek Abdel-Raheem, Hossam Ahmed, Ahmed Salem, Alpesh Doshi, Amr Abdel Raheem

**Affiliations:** 1 Dermatology and Andrology, Assiut University, Assiut, EGY; 2 Medical and Surgical Andrology, Cairo University, Cairo, EGY; 3 IVF London, University College Hospital, London, GBR

**Keywords:** intracytoplasmic sperm injection (icsi), reproductive medicine, intracytoplasmatic morphologically selected sperm injection (imsi), assisted reproduction (art), infertility

## Abstract

Objective

To differentiate the in vitro fertilization (IVF) outcomes between the two procedures, intracytoplasmic morphologically selected sperm injection (IMSI) and intracytoplasmic sperm injection (ICSI) in terms of relation to chemical pregnancy percentage, clinical pregnancy, live birth, miscarriage, and fertilization rates, respectively.

Patients and methods

This Open Prospective clinical trial was conducted during the period between Jan 2016 and Dec 2017 at one IVF unit. A total of 446 ICSI cycles and 79 IMSI cycles were conducted. Females were divided into four subgroups according to age.

Results

The study involved 525 couples (446 first trial ICSI cycles) and (79 first trial IMSI cycles). ICSI was statistically better than the IMSI in relation to the chemical pregnancy, clinical pregnancy (CPR), live birth (LBR), and fertilization rates, respectively (p < 0.05). However, there were no statistically significant differences between the ICSI and IMSI in relation to the miscarriage rate. There were statistically significant differences favoring ICSI in all subgroups except 35-37, in relation to chemical pregnancy; and in the 38-40 and >40 subgroups in relation to CPR. There were no statistically significant differences in these subgroups regarding the live birth, miscarriage, or fertilization rates.

Conclusions

This study showed that IMSI is not superior to conventional ICSI at the first attempt. Based on the findings in this study, we would not advise couples to choose IMSI at their first treatment attempt.

## Introduction

Ten percent (10%) of couples look for a reason for their infertility. The American Society of Reproductive Medicine has mentioned that the reasons for infertility are related to males (one-third of the reasons ), females (another third), and idiopathic or a mix between the two reasons(the remaining percentage) [1]. Currently, assisted reproductive technology (ART) is the gold standard for the management of infertility. It enhances the chances of conception by two mechanisms of action: first of all, it facilitates the interaction between spermatozoa and oocytes, and second, it overcomes seminal abnormalities, such as reduced number, motility, or increased morphological defects of spermatozoa [2-3]. Sperm morphology has a leading role in determining fertility and was concluded to enhance the results of fertilization and pregnancy rates in the natural fertilization process as well as in intrauterine insemination and in conventional in vitro fertilization (IVF) treatments. It is also important for the penetration of the sperm through the zona pellucida and fusion with the plasma membrane of the oocyte. Morphological abnormalities of the sperm head are associated with low fertilization, implantation, and pregnancy rates [1].

The progress in the treatment of infertility has been improved rapidly after the induction of intracytoplasmic sperm injection (ICSI) procedures [4]. Micromanipulation techniques of the ICSI have overcome the obstacles related to the patients whose semen samples were insufficient for intrauterine insemination or in vitro fertilization techniques and raised the success rates of achieving pregnancy.

ICSI represents the management-line of choice for many patients and can achieve a success rate of pregnancy up to 30% in those who have male reasons for infertility [5-6]. The power of ICSI has reached a point that the morphology of the selected sperm for injection was of secondary importance, which has increased the challenges to bypass fertilization failures in IVF procedures [7]. The selection process before the micro-injection of a motile, normal-looking spermatozoon into the oocyte’s cytoplasm usually happens under a low magnification (×200/400) that could be responsible for the underestimation of possible subtle sperm organelle malformations. A spermatozoon classified as “normal” after a morphological evaluation at low magnification could find relevant ultra-structural defects that could interrupt the fertilization method and embryo progress [8].

In 2002, Bartoov et al. [9] discovered a way of human spermatozoa assessment called “motile sperm organelle morphology examination” (MSOME). It enhances spermatozoa evaluation at high magnification (>6000x) compared to the 200-400x observed by conventional ICSI using an inverted microscope equipped with Normarski interference contrast optics. The trials of this discovery to patients undergoing conventional IVF/ICSI have led to the development of the intracytoplasmatic morphologically selected sperm injection (IMSI) [9].

The paper aims to differentiate the IVF outcomes between the two procedures, IMSI and ICSI, in terms of relation to the chemical pregnancy percentage, clinical pregnancy, live birth, miscarriage, and fertilization rates, respectively.

## Materials and methods

**Figure 1:**
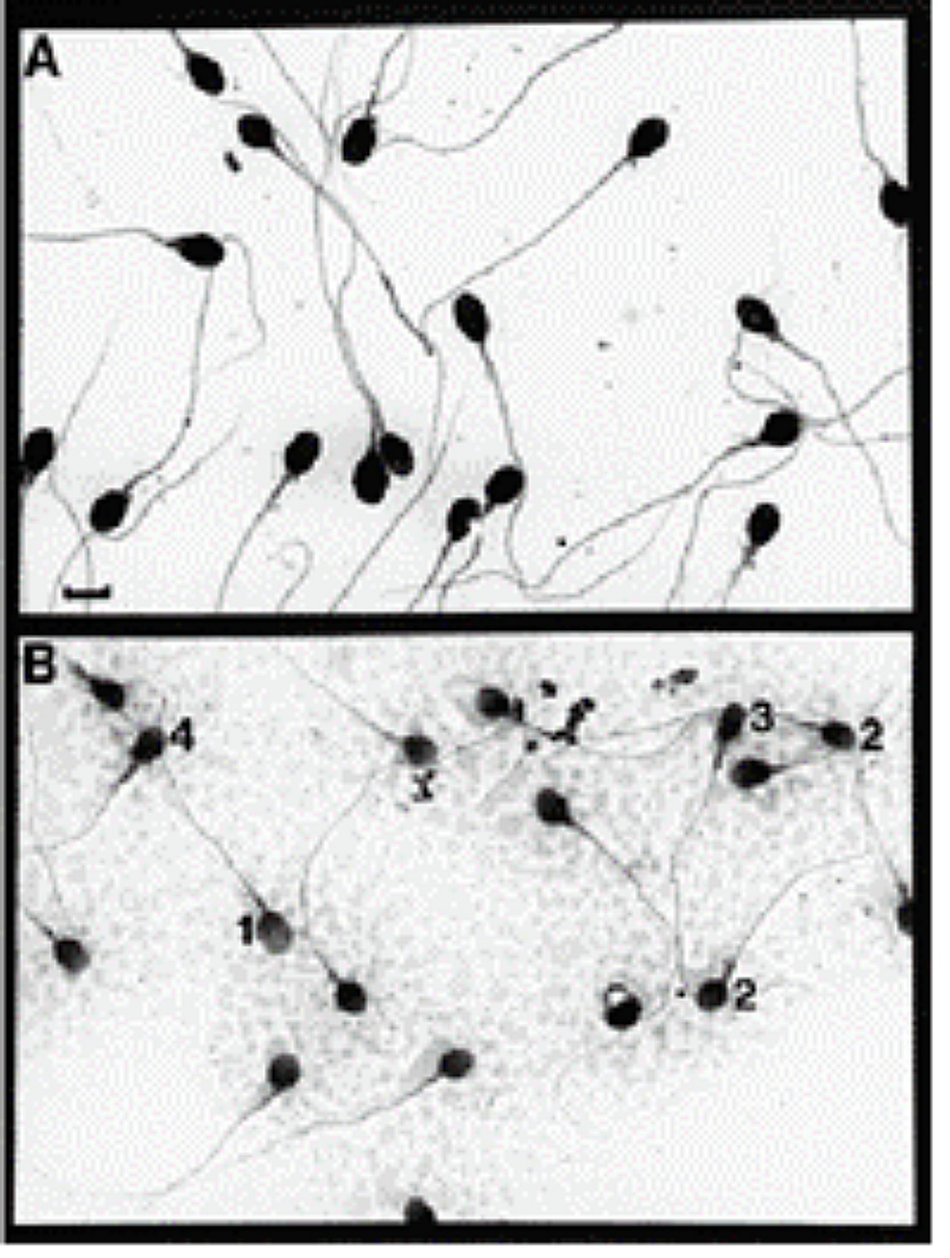
Microscopic examination of raw semen (A) Normal sperm morphology; (B) Poor sperm morphology The numbers in the figure indicate spermatozoa with: (1) normal oval head; (2) small heads; (3) a tapered head; and (4) with a small acrosome. Scale bars ϭ 3.6 μ m

**Figure 2:**
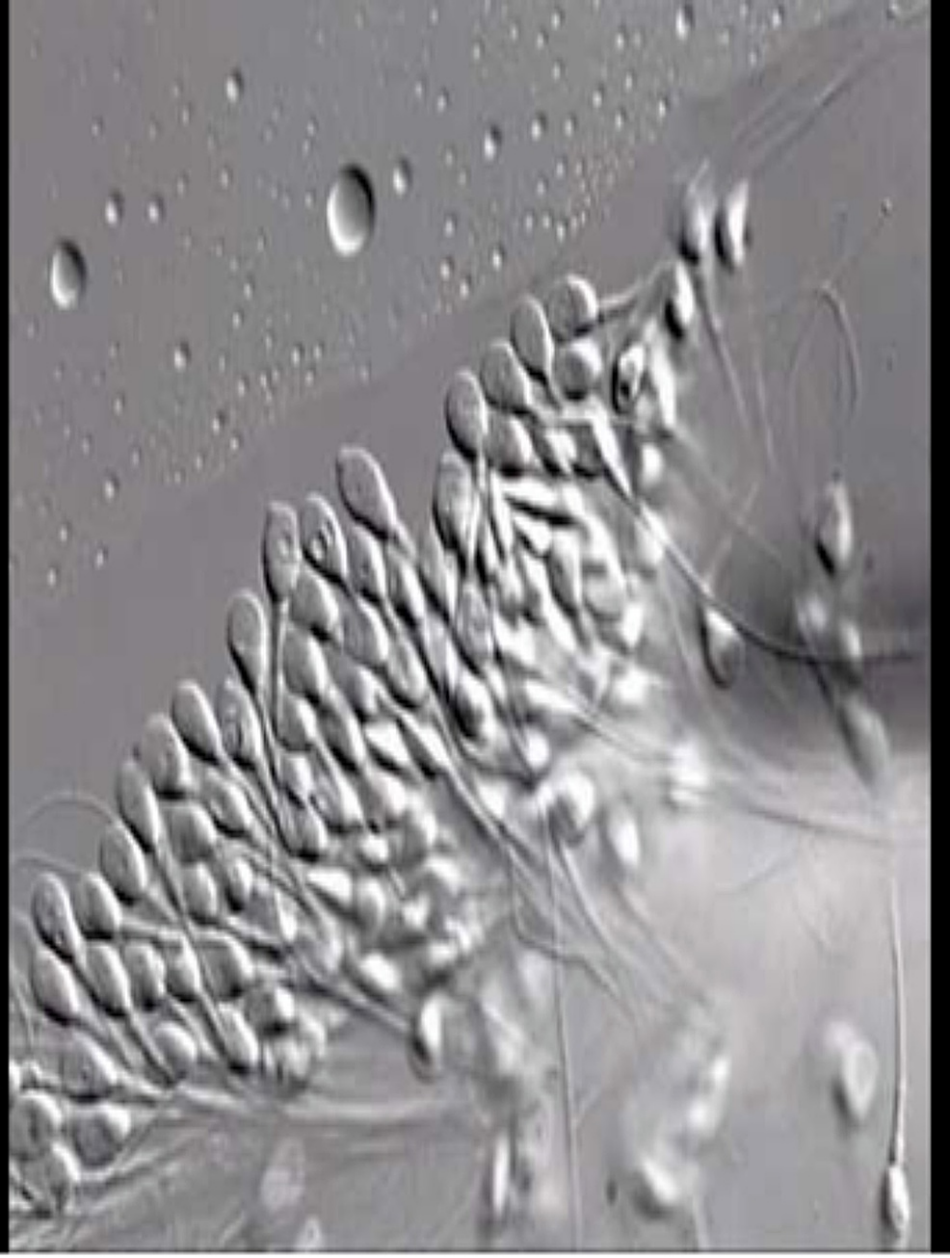
IMSI picture showing the magnification of sperms to 6000x for morphology assessment IMSI: intracytoplasmic morphologically selected sperm injection

Figures 1-2 show microscopic sperm evaluation at the conventional 200-400x and at high magnification (>6000x).

Ethical approval and consent

The proposed abstract was revised including the study protocol and approved by the ethical committee at Cairo University after fulfilling all the requirements requested for an open prospective trial, including the acceptance of all intervention techniques.

This open prospective clinical trial was conducted during the period between January 2016 and December 2017 at one IVF unit. The sample size thus was one of convenience that was formed of the consecutive patients that presented to the center during that period of time. A total of 446 ICSI cycles and 79 IMSI cycles were conducted.

Inclusion criteria included couples with primary infertility seeking fertility treatment for male factor, female factor, or combined male/female factor infertility. Only data from the first trial ICSI or IMSI cycles were included. Couples with previous failed ICSI or IMSI trials, couples using donor sperm, and couples with secondary infertility were excluded.

Male preparation

Patients were asked to give an ejaculated sample after a period of no sexual intercourse for two to five days. The sample was left for 15-60 minutes to be liquified and then assessed by IVF. In some infertile men, cryopreserved sperm was used after thawing. Semen evaluation was conducted according to standard WHO guidelines of 2010 [10].

Female preparation

Controlled ovarian stimulation was performed following the three basic elements of stimulation: gonadotrophins to stimulate multi-follicular development; gonadotropin-releasing hormone (GnRH) agonist, or antagonists to suppress pituitary function and prevent premature ovulation and triggering of final oocyte maturation 36 to 38 hours prior to oocyte retrieval using human chorionic gonadotropin (HCG).

Steps of ICSI

Sperm was selected for microinjection (using a magnification of ×400). Head defects at the spermatozoa were considered an exclusion. The oocyte injection procedure was performed at ×200 magnification using Hoffman contrast (same for both ICSI and IMSI).

IMSI procedure

Sperm selection for microinjection has been achieved at a magnification of ×6000. An aliquot of sperm preparation was dropped in a glass-bottomed dish (WillCo-dish, WillCo Wells BV, Amsterdam, The Netherlands) and assessed by the Nomarski interference contrast microscopy with a Leica DFC-280 camera (Leica Microsystems, Nanterre, France) added on a Leica DMI 6000 microscope, with an immersion objective lens ×100 and camera magnification ×1. The spermatozoon with the smallest relative vacuole area was potentially chosen. If available, spermatozoa without vacuoles was a better option for injection.

Steps of embryo culture

The injected oocytes were added to a four-well dish containing 50 μl of culture medium (G1Plus, Vitrolife, Göteborg, Sweden) and then hidden with mineral oil (FertiCult Mineral Oil, Fertipro, Beernem, Belgium). Oocyte fertilization was assessed the next day, 16-20 hours after microinjection. Embryo quality was checked the second day according to the classification system of Giorgetti [11], whereby embryos that scored a score of 3 or 4 were selected as a good morphology.

The following data were collected: Chemical pregnancy, clinical pregnancy (CPR), live birth (LBR), miscarriage (MR), and fertilization (FR) rates. Female patients were divided into four age groups: (<35, 35-37, 38-40, and ≥ 40) and the above rates were calculated and compared. Data entry and analysis were achieved using SPSS version 23 (Statistical Package for the Social Sciences, IBM Corp., Armonk, NY). Data were shown as number, percentage, mean and standard deviation. The chi-square and Fisher Exact tests were manipulated to compare qualitative variables. The Mann-Whitney test was used to compare two quantitative variables in the case of non-parametric data. P-value was considered statistically significant at P<0.05.

## Results

**Figure 3:**
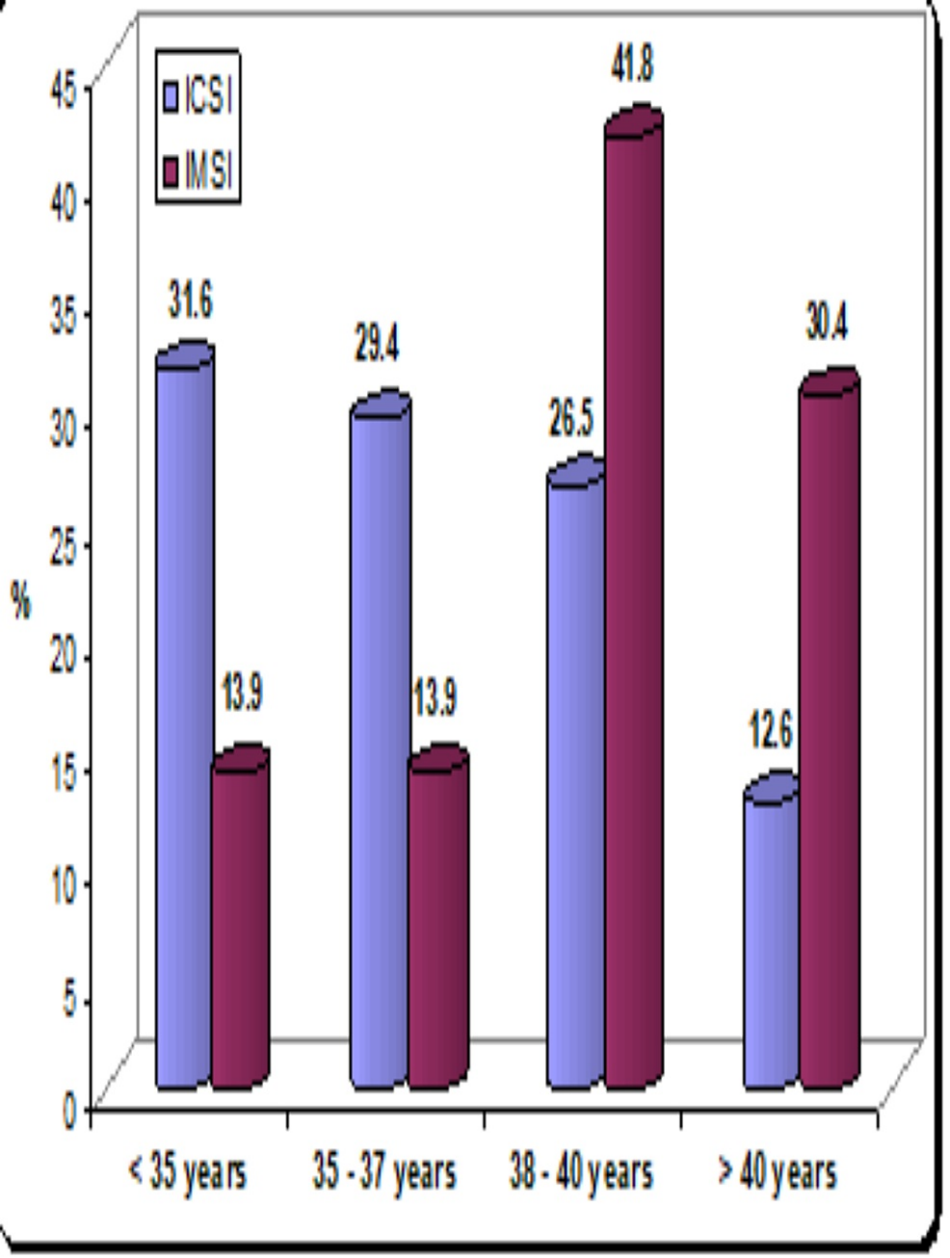
Female age distribution in different age groups in ICSI vs IMSI ICSI: intracytoplasmic sperm injection; IMSI: intracytoplasmic morphologically selected sperm injection

In this open prospective study, which was conducted in the period between January 2016 and December 2017 at one IVF unit, 525 couples were included (of whom 446 couples had ICSI and 79 had IMSI). The mean female age was 36 (20-46) and 36.85 (24-43) in the ICSI and IMSI groups, respectively. There was no statistically significant difference in the female age distribution between ICSI and IMSI groups (36.85 vs 36.04 years). Furthermore, most of the patients above 38 years had IMSI (57 out of 79 patients) (Table 1, Figure 3).

**Table 1:** ICSI vs IMSI in relation to a chemical pregnancy, fertilization rate, CPR, LBR, and miscarriage rate ICSI: intracytoplasmic sperm injection; IMSI: intracytoplasmic morphologically selected sperm injection; CPR: clinical pregnancy; LBR: live birth

	ICSI (n= 446)	IMSI (n=79)	P-value
Chemical Pregnancy	242 (54.3%)	15 (19%)	< 0.05
Fertilization Rate (Mean ± SD)	0.72 ± 0.22	0.49 ± 0.50	< 0.05
Clinical Pregnancy Rate (CPR)	189 (42.4%)	15 (19%)	< 0.05
Live Birth Rate (LBR)	170 (38.1%)	10 (12.7%)	< 0.05
Miscarriage Rate	19 (4.3%)	5 (6.3%)	0.1

The results of the data, which were statistically collected, presented that ICSI was statistically more significant than IMSI in relation to the chemical pregnancy rate (54.3 vs 19%), CPR (42.4 vs 19%), LBR (38.1 vs 12.7%), and fertilization rate (0.72 vs. 0.49%); (p < 0.05), respectively. However, there were no statistically significant differences between ICSI and IMSI in relation to the miscarriage rate (4.3 vs 6.3%) (Table 2).

**Table 2:** Comparison of female age distribution between ICSI and IMSI ICSI: intracytoplasmic sperm injection; IMSI: intracytoplasmic morphologically selected sperm injection

Age (years)	ICSI n (%) (n= 446)	IMSI n (%) (n=79)
< 35	141 (31.6%)	11 (13.9%)
35 - 37	131 (29.4%)	11 (13.9%)
38 - 40	118 (26.5%)	33 (41.8%)
> 40	56 (12.6%)	24 (30.4%)
Mean ± SD	36.04 ± 4.15	36.85 ± 5.21

When comparing the results of ICSI and IMSI in the different female age subgroups; there were statistically significant differences favoring ICSI in all subgroups except 35-37 in relation to the chemical pregnancy and in the 38-40 and >40 subgroups in relation to the CPR. There were no statistically significant differences in these subgroups regarding the live birth, miscarriage, or fertilization rates (Tables 3-7).

**Table 3:** Comparison of chemical pregnancy in different age groups in ICSI vs IMSI ICSI: intracytoplasmic sperm injection; IMSI: intracytoplasmic morphologically selected sperm injection

Age Group	ICSI	IMSI	P-value	
< 35	85 (60.3%)	2 (18.2%)	0.009*	
				35 - 37	79 (60.3%)	4 (36.4%)	0.201	
38 - 40	60 (50.8%)	9 (27.3%)	0.016*					
				> 40	18 (32%)	0	0.002*	

**Table 4:** Comparison of CPR in different age groups in ICSI vs IMSI ICSI: intracytoplasmic sperm injection; IMSI: intracytoplasmic morphologically selected sperm injection

Age (years)	ICSI	IMSI	P-value
< 35	67 (47.5%)	2 (18.2%)	0.204
35 - 37	62 (47.3%)	4 (36.4%)	0.754
38 - 40	48 (40.6%)	9 (27.3%)	0.007*
> 40	12 (21.6%)	0	0.014*

**Table 5:** Comparison of live birth rate in different age groups in ICSI vs IMSI ICSI: intracytoplasmic sperm injection; IMSI: intracytoplasmic morphologically selected sperm injection

Age (years)	ICSI	IMSI	P-value
< 35	57 (40.4%)	2 (18.2%)	0.112
35 - 37	60 (45.8%)	3 (27.2%)	0.200
38 - 40	44 (37.2%)	5 (15.1%)	0.287
> 40	9 (16.1%)	0	0.051

**Table 6:** Comparison of miscarriage rate in different age groups in ICSI vs IMSI ICSI: intracytoplasmic sperm injection; IMSI: intracytoplasmic morphologically selected sperm injection

Age (years)	ICSI	IMSI	P-value
< 35	10 (7.1%)	0	1.000
35 - 37	2 (1.5%)	1 (9.1%)	0.566
38 - 40	4 (3.4%)	4 (12%)	0.119
> 40	3 (5.3%)	0	0.550

**Table 7:** Comparison of fertilization rate in different age groups in ICSI vs IMSI ICSI: intracytoplasmic sperm injection; IMSI: intracytoplasmic morphologically selected sperm injection

Age (years)	ICSI	IMSI	P-value
	Mean ± SD	Mean ± SD	
< 35	0.71 ± 0.23	0.36 ± 0.50	0.090
35 - 37	0.71 ± 0.21	0.45 ± 0.52	0.367
38 - 40	0.69 ± 0.23	0.48 ± 0.51	0.400
> 40	0.77 ± 0.19	0.58 ± 0.50	0.881

Furthermore, male factor infertility didn’t show any significant factors between ICSI and IMSI when comparing the four semen subgroups < 5 million/ml (137 patients (30.7%) for the former technique and 34 patients (43%) for the later technique), 5 to < 10 million/ml (54 patients (12.1%) for the former technique and six patients (7.6%) for the later technique), 10 to < 15 million/ml (53 patients (11.9%) for the former technique, 10 patients (12.7%) for the later technique), and ≥ 15 million/ml (202 patients (45.3%) for the former technique and 29 patients (36.7%) for the later technique) (Table 8).

**Table 8:** Male factor numbers and percentage in relation to sperm count (million/ml) in ICSI vs IMSI ICSI: intracytoplasmic sperm injection; IMSI: intracytoplasmic morphologically selected sperm injection

Male factor (million/ ml)	ICSI (n=446)		IMSI (n=79)		P-value
	No.	%	No.	%	
< 5	137	30.7	34	43.0	0.140
5 - < 10	54	12.1	6	7.6	
10 - < 15	53	11.9	10	12.7	
≥ 15	202	45.3	29	36.7	

## Discussion

ICSI has been the golden standard management option for patients with infertility due to male reasons and low or absent success rate in the preceding IVF cycles [12]. ICSI results rely on several points, such as oocyte quality, patient’s age, and the quality of the single sperm selected to be injected into the oocyte [12]. Sperm selection has used an inverted microscope with a magnification of 200×-400×, which facilitates the assessment of both motility and normal morphology of sperms, based on evaluation of their head, neck, and tail. Normal sperm morphology is important for the success of penetration through the zona pellucida and fusion with the plasma membrane of the oocyte and morphological abnormalities of the sperm head are associated with low fertilization, implantation, and pregnancy rates [1]. However, sperm selection under low magnification only permits the detection of magnificent defects of sperm morphology [13].

As a reason for these problems with sperm selection, a new method of human sperm assessment called “motile sperm organelle morphology examination” (MSOME) was developed allowing sperm assessment at a magnification of 6000× and over, which ensures better evaluation of the acrosome, post-acrosomal lamina, neck, tail, nucleus, and mitochondria and, thus, the nomination of motile sperms with a morphologically normal nucleus and normal nuclear content and mitochondrial function. The assimilation of the MSOME technique into the ICSI procedure led to the development of intracytoplasmic morphologically selected sperm injection (IMSI) [9].

Since then, many papers have been published comparing the validity of IMSI with respect to conventional ICSI, with disagreement results. Such conflicting results have been due to the different study conduction, the heterogeneous inclusion criteria (maternal age, previous failed ICSI cycles, ovarian response, number of retrieved oocytes), and variant methods of high magnification sperm morphology classification implicated [14].

Bartoov et al. (2003) revealed that the clinical pregnancy rate after IMSI was significantly higher than that of the routine ICSI procedure (66.0% vs. 30.0%) [15]. Gonzalez et al. (2010) documented that the clinical pregnancy rate with IMSI was better than with ICSI (63% vs. 50%), although the difference was not statistically significant and the trend is clear and clinically significant in favor of IMSI [16]. The implantation rate, however, was significantly better with IMSI (44.8% vs. 29.7%). Balaban et al. (2011) concluded that IMSI did not provide a significant improvement in the clinical outcome compared with ICSI although there were trends for higher implantation (28.9% vs. 19.5%), clinical pregnancy (54.0% vs. 44.4%), and live birth rates (43.7% vs. 38.3%) in the IMSI group [17].

A meta-analysis showed that the IMSI procedure is associated with improved embryo quality, implantation, pregnancy, and miscarriage rates [18]. It is believed that the high magnification facilitates the detection of sperm-containing nuclear vacuoles. Previous research had demonstrated that sperm nuclear vacuoles are associated with sperm DNA fragmentation and, in turn, poor ICSI outcomes [19-21]. Authors also reported that the IMSI procedure is usually associated with better pregnancy rates in couples with previous and repeated implantation failures [22] and in cases with a higher degree of DNA fragmented spermatozoa [23]. In 2015, Setti et al. suggested that women of advanced maternal age (≥37) could benefit from IMSI [24]. Their results demonstrated an increase in the clinical pregnancy rate with IMSI in those women compared to conventional ICSI by nine times (4/29, 13.8% vs. 18/30, 60.0%, p<0.001).

On the other hand, Leandri et al. (2013) concluded that IMSI did not provide any significant improvement in the clinical outcomes compared with ICSI neither for implantation (24% vs. 23%), or clinical pregnancy (31% vs. 33%), nor live birth rates (27% vs. 30%) [25]. Moreover, the outcomes of IMSI were similar to the ICSI ones, regardless of the degree of sperm DNA fragmentation, nuclear immaturity, and sperm morphology. Similarly, De Vos et al., (2013) did not find any significant differences between IMSI and ICSI in the fertilization rate (79.1% vs. 77.3%) or the clinical pregnancy rate (34.4% vs. 36.7%) [26].

Our study showed that at the first treatment attempt, the IMSI technique achieved no real benefit over conventional ICSI. We concluded that there are no significant differences regarding miscarriage rate (4.3% with ICSI, 6.3% with IMSI). On the other hand, ICSI was statistically better than IMSI regarding chemical pregnancy, CPR, and LBR (54.3% and 19%, 42.4% and 19%, and 38% and 12.7%, respectively).

Our results are consistent with the findings of Gatimel et al. (2016) who found that IMSI was not preferred than ICSI when assessed as a first treatment trial in terms of implantation (12% vs 10%), clinical pregnancy (23% vs 21%), or live birth rates (20% vs 19%) [27]. And are consistent with the findings of La Sala et al. (2015) who did not conclude any significant differences between IMSI and ICSI in terms of clinical pregnancy rates [11/34 (32.3%) vs. 15/64 (23.4%)] and live birth rates [9/48 (18.8%) vs. 11/73 (15.1%)] [12].

On the other hand, a randomized trial noted the advantage of using IMSI in those patients with at least two ICSI failures previously. Antinori et al. (2008) compared 227 IMSI attempts with 219 ICSI attempts and concluded a significantly higher clinical pregnancy rate in the IMSI than in the ICSI group (39% vs 27%) and in the group with two previous ICSI failures (29.9% in the IMSI vs 12.9% in the ICSI group) [28].

Using IMSI in repeated IVF failure cycles was discussed in different studies. Klement et al. (2013) studied 1,891 IVF-ICSI cycles and 577 IVF-IMSI cycles and concluded that in the first IVF treatment, the clinical pregnancy rates were 46% and 47%, respectively, and the delivery rates were 23% and 30%, respectively [29]. In the second cycle to follow a failed ICSI, the clinical pregnancy and live birth rates were significantly higher for patients who chose to shift to the IMSI technique compared with patients who chose to go through a second IVF-ICSI cycle (56% vs. 38% and 28% vs. 18%, respectively). Berkovitz et al. (2006), however, after comparing IMSI with the previous failure of ICSI cycles with conventional ICSI found that IMSI exhibited a significantly lower pregnancy rate and a significantly higher abortion rate (18% vs. 50% and 80% vs. 7%, respectively) [30].

## Conclusions

ICSI vs IMSI has been a controversial topic in the last decade. There have been many publications on this topic, with no absolute results. Although this study has its limitations as an open prospective trial, the clinical outcome remains descriptive and there is a discrepancy in sample size between the ICSI and IMSI groups, however, our results demonstrate that for the first trial IVF treatment, IMSI has no advantage over ICSI. Based on the findings in this study, we cannot advise couples to have IMSI treatment at the first attempt. Further multicenter studies are warranted in order to reach an agreed protocol and guidelines.
